# DNA Damage Response in Cancer Therapy and Resistance: Challenges and Opportunities

**DOI:** 10.3390/ijms232314672

**Published:** 2022-11-24

**Authors:** Dana Jurkovicova, Christiana M. Neophytou, Ana Čipak Gašparović, Ana Cristina Gonçalves

**Affiliations:** 1 Department of Genetics, Cancer Research Institute, Biomedical Research Center, v.v.i. of the Slovak Academy of Sciences, 845 05 Bratislava, Slovakia; 2 Department of Life Sciences, European University Cyprus, Nicosia 2404, Cyprus; 3 Division of Molecular Medicine, Ruđer Bošković Institute, HR-10000 Zagreb, Croatia; 4 Laboratory of Oncobiology and Hematology (LOH), University Clinic of Hematology, Faculty of Medicine University of Coimbra (FMUC), University of Coimbra, 3000-548 Coimbra, Portugal; 5 Group of Environment Genetics and Oncobiology (CIMAGO), Coimbra Institute for Clinical and Biomedical Research (iCBR), Faculty of Medicine University of Coimbra (FMUC), University of Coimbra, 3000-548 Coimbra, Portugal; 6 Center for Innovative Biomedicine and Biotechnology (CIBB), 3004-504 Coimbra, Portugal

**Keywords:** DNA damage response, drug resistance, DNA damage repair inhibitors, biomarkers

## Abstract

Resistance to chemo- and radiotherapy is a common event among cancer patients and a reason why new cancer therapies and therapeutic strategies need to be in continuous investigation and development. DNA damage response (DDR) comprises several pathways that eliminate DNA damage to maintain genomic stability and integrity, but different types of cancers are associated with DDR machinery defects. Many improvements have been made in recent years, providing several drugs and therapeutic strategies for cancer patients, including those targeting the DDR pathways. Currently, poly (ADP-ribose) polymerase inhibitors (PARP inhibitors) are the DDR inhibitors (DDRi) approved for several cancers, including breast, ovarian, pancreatic, and prostate cancer. However, PARPi resistance is a growing issue in clinical settings that increases disease relapse and aggravate patients’ prognosis. Additionally, resistance to other DDRi is also being found and investigated. The resistance mechanisms to DDRi include reversion mutations, epigenetic modification, stabilization of the replication fork, and increased drug efflux. This review highlights the DDR pathways in cancer therapy, its role in the resistance to conventional treatments, and its exploitation for anticancer treatment. Biomarkers of treatment response, combination strategies with other anticancer agents, resistance mechanisms, and liabilities of treatment with DDR inhibitors are also discussed.

## 1. Introduction

DNA and other biological molecules are susceptible to damage, but DNA damage can have much more complex consequences due to its function. Unlike other molecules, which are synthesized and degraded depending on their necessity, DNA is constantly present and replicating when the cell is in division. Therefore, there is a great need for a reparation system to maintain DNA integrity. Estimations suggest that every day, about 10^5^ lesions occur in the cell [1]. In addition to exogenous threats, such as irradiation, chemical pollutants, and chemical agents, endogenous processes increase reactive oxygen species (ROS), damaging DNA directly or indirectly. DNA damage includes single- and double-strand brakes, inter-and intra-strand links, abasic sites, bulky adducts, and base changes, such as 8-deoxyguanosine [1]. Due to the diversity of DNA damage, there are different repair mechanisms implicating many proteins. The activation of different repair mechanisms with the primary goal to restore DNA integrity is collectively known as the DNA damage response (DDR). These proteins/genes were historically identified in Fanconi’s anemia (FA), a rare genetic disorder characterized by bone marrow failure, skeletal malformation, and increased cancer incidence. Mutations in this rare disease include genes in the FA pathway that are fundamental genes involved in DNA damage repair [2]. If DNA damage is not repaired or misrepaired, genomic instability and mutations will be established, which are among the hallmarks of cancer [3]. The DDR plays a relevant role not only in cancer development but also in cancer treatment. Defects in DDR genes are known as cancer drivers, and cells with deficient DDR show a higher sensitivity to DNA-damaging agents [4]. In this review, we summarize recent evidence of DDR pathways in cancer therapy, its role in the resistance to conventional treatments, and its exploitation for anticancer treatment. Since DDR is involved in cancer development and is a molecular target of cancer treatment, biomarkers of treatment response, combination strategies with other anticancer agents, resistance mechanisms, and liabilities of treatment with DDR inhibitors are also discussed.

## 2. The DNA Damage Response

The DNA damage response pathways are composed of an intricate system of sensors, transductors, and effectors involved in DNA repair and cell cycle checkpoint control that manage the execution of DNA replication and cell proliferation. The wide variety of DNA lesion types requires multiple and different DNA repair mechanisms. To repair single-strand breaks (SSBs), the mismatch repair (MMR), base excision repair (BER), and nucleotide excision repair (NER) are activated, while homologous recombination (HR) and non-homologous end joining (NHEJ) pathways repair the double-strand breaks (DSBs) [5]. DDR also includes damage tolerance processes and the consequent signaling control of cell decisions on senescence or death. In parallel, DDR affects epigenetics and gene expression regulation preferentially related to the induction of apoptosis [6,7].

### 2.1. Single-Strand Break Repair

The need for MMR occurs during DNA replication, where polymerases are prone to mistakes. Therefore, a mismatch repair system repairs wrongly matched bases to obtain replicative fidelity. The mismatch repair system in humans includes several proteins: DNA mismatch repair protein Mlh1 (MLH1), DNA mismatch repair protein Msh2 (MSH2), DNA mismatch repair protein Mlh3 (MLH3), DNA mismatch repair protein Msh6 (MSH6), DNA mismatch repair endonuclease PMS2 (PMS2), and DNA mismatch repair protein Msh3 (MSH3) [8,9]. These proteins act as heterodimers MSH2 with MSH6 or MSH3 (MutSα or MutSβ complexes, respectively) and MLH1 with PMS2 or MLH3 (MutLα, MutLβ, or MutLγ complexes, respectively) [9]. In addition to these protein complexes, proliferating cell nuclear antigen (PCNA) with the help of MSH2/MSH3 and MSH2/MSH6 complexes recognizes and binds to the mispaired region [10]. Furthermore, single-strand DNA-binding protein RPA (replication protein A) and EXOI (exonuclease I) both contribute to MMR by protecting the gap from excision, while EXOI is needed for the repair of the break located either 5′ or 3′ to the mispair [11]. The evidence strongly suggests that polymerase δ (pol δ) is required for MMR [12], and DNA ligase I is needed for the final step in MMR [13].

NER is the mechanism of bulky adduct reparation. The offset of this mechanism can be initiated by global genome NER or transcription-coupled NER [14]. Like other repair systems, there are two steps involved: recognition of the damage and the reparation step. Bulky adducts cause DNA distortions recognized by XPC-RAD23B, but only if the nucleotide opposing the lesion is not missing [15]. Once XPC-RAD23B recognizes the bulky adduct, a small bubble is formed, and TFIIH, a complex of 10 proteins, is recruited [16]. Then, XPB translocase and XPD helicase open the bubble even more (22 to 25 base pairs) [17], which allows binding of XPA and RPA. RPA protects the undamaged strand while XPA verifies the damage and further recruits XPF-ERCC1 endonuclease that cleaves the 5′ end, while XPF cleaves the 3′ end [18]. The resulting gap is filled by DNA polymerase δ and ε, replication factor C (RFC), PCNA, and RPA [19].

Like NER, BER can be activated during transcription and initiated by global genome BER. The damaged base is recognized and removed by DNA glycosylase [20]. Several DNA glycosylases recognize damaged bases. Monofunctional DNA glycosylases, such as alkyl adenine DNA glycosylase or uracil DNA glycosylase, create abasic sites, while bifunctional DNA glycosylases, such as 8-oxoguanine DNA glycosylase (OGG1), NEIL1−3, additionally cleave the 3′ site on the abasic sugar [21,22]. The excision of the damaged base initiates the repair process, where the excision site is recognized by apurinic/apyrimidinic endonuclease (APE) [23]. If monofunctional DNA glycosylase acts on the damaged nucleotide, APE1 cleaves the site, while after bifunctional glycosylase, APE2 is the endonuclease responsible for the cleavage [23]. Replacement of the damaged/missing nucleotide is filled by the action of polymerase β or, if the gap is bigger than one nucleotide, with polymerase δ or ε. The ends are ligated by DNA ligase I (LIG1) or DNA ligase III (LIG3) [24]. Alternative pathways include poly(ADP-ribose) polymerase 1 (PARP1), which acts as a sensor for the damaged nucleotide [25]. PARP1, a protein located in the nucleus in high abundance, takes part in the BER system [26]. Following SSB or DSB DNA damage, PARP1 swiftly localizes to the damaged site, and its enzymatic activity is increased 10- to 500-fold. This activity leads to the synthesis of poly-ADP-ribose (PAR) chains after damage within 15 to 30 s [26]. PARP1 transfers 50–200 residues of PAR to itself and its substrates, including enzymes such as DNA polymerases, topoisomerases, and DNA ligase-2, as well as histones, high-mobility-group proteins, and transcription factors [27]. The modification of these proteins by poly (ADP-ribosyl)ation (PARylation) allows PARP1 to control not only cellular repair, DNA replication, and transcription but also protein degradation, organization of the cytoskeleton, and other cellular functions [27]. The extent of PARP1 activation after DNA damage controls whether cells will live or die. Caspase-3 and -7 mediate cleavage of PARP1 into a ∼25-kDa N-terminal and a ∼89-kDa C-terminal fragment, which are among the hallmarks of apoptosis. Cleaved PARP1 cannot participate in DNA repair during apoptosis and allows cells to commit to the apoptotic pathway [28,29]. Nevertheless, PARP1, if excessively engaged, induces cytotoxicity. Therefore, the action of DNA repair protein XRCC1 (XRCC1) is needed since XRCC1 binds PARP1 and DNA ligase II, forming a complex, which controls the activity of PARP1 and prevents its toxicity [30]. ROS and the dysregulation in the activity of DNA topoisomerase 1 (TOP1) create SSBs, resulting in abortive TOP1–DNA complexes, which are removed by tyrosyl-DNA phosphodiesterase 1 (TDP1), a target of PARP1 [31]. TDP1 thereby enhances the recruitment of other proteins involved in the repair [32]. Furthermore, TDP1 and PARP1 recruit XRCC1, another substrate for PARP1 [31]. PARylation of XRCC1 recruits polymerase β and DNA ligase III, which finalize the repair [31].

### 2.2. Double-Strand Breaks Repair

Double-strand breaks are repaired by at least five different mechanisms: canonical non-homologous end joining (cNHEJ), HR, alternative non-homologous end joining (Alt-NHEJ), single-strand annealing (SSA), and break-induced replication (BIR) [33]. The first step is the recognition of the break by scaffolding proteins 53BP1 and BRCA1, after which the break is repaired by one of the mentioned mechanisms [33]. In addition, PARP1 can also detect double-strand breaks [34]. However, the repair pathway choice is regulated by resectosome, a protein complex responsible for DNA end resection [35]. Resectosome comprises a helicase, a nuclease, and other regulating proteins, while the length of the resected DNA determines the pathway [36]. Canonical NHEJ joins two ends of the break without homology check [36], while HR uses the sister chromatid as a template for repair [37], thereby dictating the part of the cell cycle where each of these two mechanisms can function and limiting the HR to S/G2 transition. Due to the template, HR is mostly error-free in its outcome as the newly synthesized chromatid or the homologous chromosome is used as a template for DNA repair [38,39]. Still, in some cases, it can cause genetic instability and rearrangements [37].

Both c-NHEJ and HR repair start with binding Ku70-Ku80 heterodimer to a double-strand break [40]. The binding of Ku70-Ku80 recruits other factors: DNA-dependent protein kinase catalytic subunit (DNA-PKcs), DNA ligase IV (LIG4), and the associated scaffolding factors of DNA repair protein XRCC4 (XRCC4), XRCC4-like factor (XLF), and paralogue of XRCC4 and XLF (PAXX), which bring the two ends closer, enabling end processing by Artemis and DNA polymerases λ and μ [37]. The HR mechanism is usually associated with cancer and BRCA1 and BRCA2 mutations, which are linked to hereditary breast and ovarian cancer [41]. However, new evidence suggests that the basis of hypersensitivity of BRCA-deficient tumors is not double-strand breaks induced by chemotherapy but rather single-strand breaks [42]. The HR process includes numerous steps, the first one being recognition by two kinases, ATM and ATR, which phosphorylate targets: CHEK2, P53, BRCA1, and H2AX. BRCA1 serves as a scaffold that recruits other proteins [43]. Recognition is followed by DNA resection with MRN complex together with EXO1 and the recQ-like DNA helicase (BLM) heterodimer [43]. The endonuclease activity causes displacement of Ku70-Ku80, and binding of RPA occurs to the single-stranded-DNA [37]. Next, BRCA2 working with PALB2 loads RAD51 to the single-stranded DNA and mediates strand exchange using the sister chromatid as a template [37,43,44].

### 2.3. Epigenetic Control in DNA Damage Response

To properly understand how cells control complex DDR, epigenetics and miRNA regulations cannot be omitted. Epigenomic alterations are known to significantly affect gene expression and overall tumor heterogeneity. Therefore, it is not surprising that DNA repair processes are also affected by epigenetic chromatin regulation. Histone deacetylases (HDACs) are important players in chromatin preparation to promote DSBs repair through HR and NHEJ. For example, PARP1 recruits the nucleosome remodeling deacetylation (NuRD) complex by attaching a PAR chain signal essential for DSB repair [45]. In fact, PARylation inhibition stops chromatin relaxation at DNA damage sites, suggesting that chromatin relaxation is PARylation-dependent [46]. On the other hand, HDAC1 and HDAC2 deacetylases stimulate the RNF8/RNF168-dependent ubiquitination at DSB promoting NHEJ repair, while histones H4 and H2 acetylation/deacetylation, at specific sites, switch DNA repair from NHEJ to HR via 53BP1 binding regulation at the DSB site [45,47]. DNA methylation is a common and stable epigenetic mechanism of gene inactivation, and in cancer cells, DDR components also show changes in their gene promoter methylation status [48]. For example, hypermethylation of *OGG1* genes was observed in thyroid cancer [49], *MLH1* gene in oral squamous cell carcinoma [50], neck squamous cell carcinoma [51], non-small cell lung cancer (NSCLC) [52], acute myeloid leukemia (AML) [53], gastric cancer [54], ovarian cancer [55], and *BRCA1* gene in breast cancer [56], bladder cancer [57], NSCLC [58], and gastric cancer [59]. Additionally, the methylation status of some DDR genes has been used as diagnostic, prognostic, and therapy response biomarkers in various cancer types. *MLH1* methylation has been indicated not only as a diagnostic biomarker and an indicator of good prognosis in several cancers, including colorectal, ovarian, and breast cancers, but also as a therapy response biomarker associated with platinum compounds, temozolomide, and epirubicin resistance and with methotrexate sensitivity [60].

MicroRNAs (miRNAs) are other plausible players important for DDR modulation. MiRNAs regulate multiple processes of tumorigenesis, post-transcriptionally controlling expression of components of DNA damage repair and other mechanisms defining response to treatment and overall outcome and survival of cancer patients. As regulatory elements, miRNAs can modulate cancer cell sensitivity toward DNA-damaging agents by regulating the expression level of DNA repair genes. Therefore, miRNAs represent promising therapeutical tools modifying treatment response, mainly in highly resistant cancers, such as breast cancer [61,62,63,64,65]. In DDR, miRNAs play a significant regulatory role as transcriptional and post-transcriptional regulators of DNA damage sensors, signal transducers, and effector genes. For example, miRNAs can directly target genes involved in cell cycle regulation, e.g., miR-34 targeting cyclins, miR-93 targeting *E2F1* and *CCND1* [66,67], or miR-125b and miR-34a controlling expression of *TP53* [61]. DNA repair checkpoints are targets of, for example, miR-15, miR-195, and *CHK1* and *WEE1* are targets of miR-497 [68,69,70]. MiR-191 was shown to target *CHK2* in osteosarcoma cells [71]; miR-124 targeting *PARP1* [72]; miR-181a/b targeting *ATM* [73]; miR-182 and miR-218 targeting *BRCA1* [74,75]; and miR-155, miR-103/miR-107, and miR-221/222 targeting DDR gene *RAD51* [61,76]. MiR-494 and miR-99b were upregulated after γ-irradiation and directly inhibited a protein complex consisting of MRE11, RAD50, and NBS1 (MRN complex) crucial for DSBs repair in human endothelial cells [77]. In aggressive triple-negative breast cancer (TNBC), miR-155 [78] and miR-21 [79] act as typical oncomiRs, and miR-205 [80,81], miR-200c/miR-141 [82], let-7 [83], and miR-221/222 [84] were shown to be typical tumor suppressor miRNAs. MiR-19a-3p, miR-218-5p, and miR-874-3p were shown to directly target *RAD51* and *BRCA2* in HR and *XRCC5* (*KU80*) and *PRKDC* of the NHEJ pathway, affecting recombination repair. XRCC5 can also be targeted by miR-526b and miR-623 to induce apoptosis when overexpressed in NSCLC and breast cancer cells [85,86]. Today, there is no doubt that miRNAs importantly regulate the expression of components of DNA repair pathways. The exploitation of DDR gene/miRNA interactions and the possibility of their easy inhibition with antagomiRs or reintroduction using miRNA mimics open a novel field for clinical utilization in terms of new potential biomarkers and new therapeutic tools.

## 3. DNA Damage Response Inhibitors in Cancer Therapy

The DDR mechanisms are involved in the control of core processes of cell fate, survival, and genome maintenance. In cancer, accumulated genetic defects compromise the cell response to physiological growth control and promote uncontrolled division and evasion of apoptosis. Vogelstein and collaborators identified approximately 130,000 different mutations in more than 3000 individual drivers of tumorigenesis, including both oncogenes and tumor suppressors. From these, about 330 genes were identified as drivers involved in the regulation of cell survival, genome maintenance, and overall DDR. These genes are valuable targets for new approaches to cancer treatment [87].

The effect of primary anticancer therapies, including ionizing radiation and different chemotherapeutic agents that damage both nuclear (nDNA) and mitochondrial (mtDNA) DNA, are expected to drive the cancer cell, directly or indirectly, towards death. Cisplatin represents such a highly efficient DNA-damaging agent with substantial anticancer effects. Despite the discovery of its cytotoxic effects and its first Food and Drug Administration (FDA) approval for treating testicular cancer in 1978, cisplatin is still used as first-line chemotherapy in numerous solid tumors. The cell response to cisplatin is complex and includes mechanisms regulating its entry, exit, accumulation, and detoxification and mechanisms modulating DNA repair, cell survival, and the tumor microenvironment [88].

### 3.1. DNA Damage Response Inhibitors in Single-Agent Approaches

Cancer cells show a higher level of endogenous DNA damage and increased replication stress than normal cells and usually have one or more DDR pathways disabled. Such deficiencies are attractive points for novel cancer treatments development, mostly those exploiting synthetic lethality concepts [89,90]. In principle, synthetic lethality in cancer treatment is based on targeting and inhibiting the DDR pathway that is left functional. If one DDR pathway is compromised and not functional, e.g., due to mutations in one or more DNA repair genes, the cancer cell attempts to restore the DNA damage utilizing the backup repair mechanism. However, if this backup mechanism is pharmacologically targeted, the cancer cell has no functional DNA repair pathway and is doomed. In 2014, in both Europe and the USA, Olaparib—a PARP inhibitor (PARPi)—was the first DDR inhibitor (DDRi) approved for cancer treatment [89]. Shortly after, in 2017, two other PARP inhibitors, Rucaparib and Niraparib, were FDA-approved for use in cancer patients with *BRCA* mutation as well as for non-carriers of *BRCA* mutations to treat primary peritoneal cancer, fallopian tube, or recurrent epithelial ovarian cancer resistant to cisplatin chemotherapy [91,92]. Later, Talazoparib was approved by the FDA to treat patients with germline *BRCA* (g*BRCA*) mutations or metastatic breast cancer [93,94]. In 2020, the FDA approved Veliparib for use in combination with gamma-ray radiotherapy or chemotherapy for advanced lung squamous cell carcinoma [95] and in combination with paclitaxel/carboplatin for the treatment of recurrent ovarian, breast, and lung cancer patients [96,97,98]. Veliparib and another PARPi Iniparib in combination with gemcitabine/carboplatin for breast and lung cancer treatment reached phase III trials [99].

In clinical practice, the conventional treatment of women with OC is based on debulking surgery and selecting first-line platinum-based chemotherapy, followed by second-line platinum chemotherapy in case of relapse. Further management of OC patients diverges with the decision for maintenance treatment or active surveillance watching until the third relapse [100]. If OC patients harbor BRCA mutation, targeted therapy with PARPi may replace chemotherapy to maintain the response, delay disease progression, and prolong the period between treatment cycles [91,101]. Recently, Olaparib has been approved for maintenance treatment in the first-line setting for women with a BRCA mutation [102]. Similarly, rucaparib was approved in the treatment setting for patients with relapsed BRCA-mutated platinum-sensitive OC [103] and has been shown to be beneficial in both the maintenance as well as treatment settings. The substantial benefit of PARPis in the first-line setting has been demonstrated in randomized phase III trials (SOLO-1, PAOLA-1, PRIMA, VELIA) [104,105,106,107]. However, besides the significant contribution of PARPis to better treatment management of OC patients, the side effects of PARPis on patients’ quality of life must be thoroughly monitored.

PARP is the best-known element of the DDR. Functional PARP identifies single-strand breaks and utilizes nicotinamide adenine dinucleotide (NAD^+^) to form poly ADP-ribose chains that open chromatin to allow DNA repair proteins to access the DNA [108]. PARPi prevents the formation of PAR chains and keeps PARP on the DNA at SSBs. Consequently, formed PARP–DNA complexes stall or collapse the replication fork and generate DSBs. DSBs can be repaired by HR, but if HR is missing or defective in cancer cells, as in *BRCA1* mutation-carrying cancers, the cell must use error-prone NHEJ, leading to genomic instability and cancer cell death [108,109,110]. PARPs are involved in the repair of SSBs through BER and DSBs, through HR, NHEJ, and alt-NHEJ (or microhomology-mediated end joining, MMEJ). Along with successful validation on patients carrying *BRCA1* and *BRCA2* mutations [111], positive effects of PARPi were also observed in patients without *BRCA* mutations with high-grade serous or poorly differentiated ovarian carcinoma or TNBC [112]. Accordingly, PARP inhibition as a therapeutic approach successfully expanded to other cancers, including pancreatic, endometrial, prostate, urothelial, colorectal, lung, and glioblastoma [113]. Overall, DDR involves more than 450 proteins [89,114,115], and several are being investigated as potential novel therapeutic targets. The most promising include DNA damage sensors (MLH1), damage signaling molecules (ataxia telangiectasia mutated (ATM), ATM- and RAD3-related (ATR), CHK1, CHK2, DNA-dependent protein kinase, catalytic subunit (DNA-PKcs), and WEE1), or effector proteins for DNA repair (POLQ, RAD51, or PARG) [113]. Several DDRi are currently in preclinical and clinical trials (Table 1). Clinical trials have been initiated to test their targeting with single agents or in combination therapy. In parallel, different technologies are being explored to screen for synthetic lethal combinations, including small interfering RNA (siRNA) or exploiting CRISPR-Cas9-based strategies to target them for anticancer treatment purposes.

### 3.2. DNA Damage Response Inhibitors in Combinatory Therapies

To gain maximum efficiency of anticancer treatments, combinations of individual agents and strategies are tested and validated. Concerning the interplay between epigenetics and DNA repair, the most explored therapeutic approach is the combination of epigenetic inhibitors with chemotherapeutic agents. Additionally, several clinical trials have been initiated to evaluate the efficacy of combined administration of HDAC and PARP inhibitors. Such combinations have been examined in pre-clinical models and effectively kill prostate, ovarian, and breast cancer cells [116,117,118,119]. Similarly, combined administration of DNMT1 and PARP in AML and breast cancer showed a synergistic activity [120]. Chromatin remodeling inhibitors also prevent HR repair and sensitize different cancers cells towards DNA-damaging agents [121], while HDAC, DNMT, and LSD1 inhibitors restore chemosensitivity in different solid tumors [122]. A synergism of combinations has been identified between radiotherapy and epigenetic inhibitors [123,124,125,126,127]. However, due to high toxicity and limited patient benefits, these approaches still require more investigation and clinical validation. In recent years, much attention has been paid to investigating the therapeutical effect of combinations of PARP inhibitors with antiangiogenic therapy. Multi-kinase inhibitors targeting VEGFR, PDGFR, and FGFR were shown to sensitize tumor cells to PARP inhibitors via induction of hypoxia and triggering HR defects [128].

Combination regimens between two DDRi have also been investigated. The synergic effect of PARPi in combination with ATR inhibitor (ATRi) was reported in HR-deficient HGSOC in vivo [129]. The therapeutic combinatory approach of non-toxic concentrations of a CHK1 inhibitor (CHK1i; PF-00477736) with a WEE1 inhibitor (WEE1i; MK-1775) showed a synergistic effect in breast, ovarian, colon, and prostate cancer cell lines in a P53 status-independent manner [130]. The combinations of the CHK1is (PF-00477736 or AZD-7762) with the WEE1i (AZD-1775) showed a synergistic effect in all the diffuse large B-cell lymphoma cell lines independently of the molecular subtype and *MYC* status [131]. The combination of CHK1i with WEE1i also showed a strong synergy in mantle cell lymphoma [132], lung, prostate, and erythroleukemia [133]. The synergistic effect of DDRi in B-cell lymphomas was also observed in combinations of ATRi with CHK1i and ATRi with WEE1i [134]. Moreover, the combination of ATR (VE-821) and CHK1 inhibitors (AZD7762) induced replication fork arrest, ssDNA accumulation, replication collapse, and synergistic cell death in osteosarcoma, breast, and lung cancer cells in vitro and in vivo [135]. The co-administration of Olaparib and AZD1775 (WEE1 inhibitor) demonstrated a synergistic antiproliferative effect in TNBC cell lines and significantly inhibited tumor growth in a xenograft model of BC [136]. Preclinical studies showed that ATR inhibitor synergizes with WEE1i in TNBC [18,137]. This therapeutic association reduced cell proliferation and induced cell death in several BC cell lines [137,138] and tumor remission, increased survival, and inhibited metastasis in orthotopic BC xenografts mouse models [138]. Bukhari et al. also demonstrated the therapeutic potential of this association in mammospheres, reporting similar sensitivities to the combined treatment in cancer stem cells [138]. Kim and colleagues demonstrated, using acquired and de novo PARPi and platinum-resistant models, that PARPi (AZD2281) in combination with ATRi (AZD6738) synergistically decreased cell viability and colony formation using doses with minimal off-target effects [139]. This combination also induced tumor regression and a significant increase in overall survival in HGSOCs patient-derived xenograft (PDX) models.

The metabolic vulnerability of cancer cells is a highly relevant dimension that can be exploited for therapeutical targeting and potential overcoming therapy resistance. Mammalian target of rapamycin (mTOR) kinase, mTORC1, and mTORC2 complexes are considered critical drivers of cancer drug resistance that integrate signaling pathways driving cell metabolism and growth [140,141]. Both mTOR complexes belong to effectors of the most oncogenic drivers, including RAS-driven MAPK and PI3K-AKT pathways. Sustained mTOR signaling contributes to resistance to therapeutics targeted against the driving oncogenes [142] or chemotherapy resistance, for example, by inducing the FA DNA repair pathway [143] and modulating other proteins that are essential in chromosomal integrity and DNA damage response [144,145]. Deregulation of mTOR has been found in various human cancers [146], including resistant ones such as TNBCs [147,148]. Inhibitors of mTOR are therefore considered a valuable addition to chemotherapy or targeted cancer therapy, either as an option for relapsed patients or as a frontline combination therapy to prevent or delay the development of resistance due to sustained mTOR signaling [142,149].

Similarly, using DDR inhibitors and/or radiation as sensitizers provide new potential to increase immunotherapy efficacy. Immunotherapy attracts much attention and is considered a breakthrough in the field of cancer treatment. Individual DNA repair pathways’ defects were associated with immune checkpoint blockade response. DNA damage induced in cancer cells upon radiation or chemotherapy leads to the release of chromosome fragments or small pieces of DNA that activate an immune response. When DDRi (e.g., PAPRi) are used, more DNA fragments are released, making tumor cells more immunogenic and more sensitive to immunotherapy. For example, defects in MMR result in neoantigen generation [150] that is associated with better anti-PD-1/PD-L1 immunotherapy outcomes [151]. The benefits of multiple combination therapies involving immune checkpoint inhibitors with DDRi are undergoing clinical trials.

A novel, although limited, field of anticancer approaches is opened via targeting mtDNA repair pathways. The capacity of mtDNA repair significantly contributes to therapeutical cancer cell response. BER is the main repair pathway used by mitochondria to repair mainly ROS-induced lesions. mtDNA carries many mutations that usually correlate with cancer progression [152]. Defective mtDNA repair pathways or downregulated mtDNA repair-associated proteins, such as mitochondrial transcription factor A (mtTFA) and POLγ, together with administered DDR inhibitors, can result in higher sensitivity of cancer cells to radio- or chemotherapy [153,154].

## 4. Biomarkers of DNA Damage Response Inhibitors

As mentioned previously, a significant number of new DDR inhibitors and DDR-based therapeutic strategies have arisen recently. The remarkable clinical success of PARP inhibitors in patients with *BRCA1* and *BRCA2* gene mutations showed that the clinical utility of DDRi relies on establishing response biomarkers that select patients who will benefit from these therapies. PARPi has shown efficacy in HR-deficient cancers, including those with *RAD51C*, *RAD51D*, and *PALB2* mutations [155] and with a “BRCAness” phenotype [156], but not all HR alterations have the same impact on the efficacy of these inhibitors [157,158]. The “BRCAness” phenotype is defined by the lack of *BRCA1/2* mutations in tumors with similar molecular phenotypes. This phenotype can result from mutations and epigenetic modifications of HR-related genes that cause homologous recombination deficiency (HRD), such as *RAD51C*, *RAD51D*, *ATM*, *BARD1*, *PALB2*, *BRIP1*, and *MRE11* mutations and BRCA1 hypermethylation [155,156,159,160,161,162]. The sensitivity to platinum-based chemotherapy is considered a surrogate biomarker of “BRCAness” phenotype to PARPi, and FDA approved this biomarker as a response biomarker for Olaparib therapy in maintenance settings [163]. However, not all patients who respond to platinum-based therapy will respond to PARPi, and some patients resistant to these conventional therapies will respond to PARPi [164]. Moreover, gene alterations, mutations, or functional loss of proteins involved in DDR mechanisms result in defective *ATR*, *CHEK1*, *CHEK2*, *DSS1*, *MRE11A/NBS1*, Fanconi anemia complementation group (*FANC* family of genes), *EMSY*, *XRCC2*, *XRCC3,* or *PTEN*, predispose patients to the success of PARP inhibitors for cancer treatment [165,166,167,168].

Throughout the years, several HRD assays have been developed to try to identify patients who will benefit from DDRi. These tests include the mutational status of DDR genes that identify specific causes of HRD, “genomic scars” or mutational signatures that identify HRD cancers, and functional assays that provide a readout of HRD or homologous recombination proficiency [169]. HRD cancers are expected to have genomic instability, and patients with these features are identified as “BRCAness”. For example, tumors with *BRCA1/2* mutations were associated with loss of heterozygosity (LOH), large genomic deletions, large-scale transitions (LST), and telomeric allelic imbalance (TAI) [170,171,172,173,174], while microsatellite instability (MSI) is characteristic of MMR deficiency [175]. A combination of these genomic scars, LOH, LST, and TAI, robustly predicted the “BRCAness” phenotype and the sensibility of PARPi and was the basis of myChoise HDR (Myriad Genetics) and FoundationOne CDx (Foundation Medicine) commercial assays [158,176]. Additionally, a genomic mutational signature, “signature 3”, was significantly associated with *BRCA1/2* mutations [177]. The reduced nuclear RAD51 foci have been associated with *BRCA1/2* mutations and with PARPi responses. However, currently, the functional assays of HRD, as an estimation of nuclear RAD51 amount being the most used system, have insufficient evidence to establish their clinical value to predict PARPi response [169]. Several other genomic alterations have been proposed as potential biomarkers of DDRi response. For example, decreased CHK1 phosphorylation, increased expression of γH2AX, and increased replication fork instability are associated with ATR inhibitors response, while P53 deficiency and replication stress promoting genomic charges, including *CCNE1* and *MYC* amplification, are associated with WEE1 inhibitors response [158]. *KRAS* mutations and the overexpression of *CCNE1*, *CCND2*, and *MYC* genes induce hypersensitivity to ATR inhibitors in cancer cell lines [178,179,180]. Moreover, tumor cells with loss of H3K36me3 due to mutation in *SETD2*, the gene that encodes a histone lysine methyltransferase, or mutation in histone H3 itself showed HR, NHEJ, and MMR impairment and sensitivity to WEE1, CHK, or ATR inhibitors [181]. These tumors were also sensitive to PARP and ATR inhibitors [158].

According to the European Society for Medical Oncology (ESMO) Translational Research and Precision Medicine Working Group, the most useful predictive biomarkers for HRD and indicate the PARPi benefit in the clinic are single-gene aberrations and/or genomic scars [169,182]. These tests reflect HRD phenotype and aim to identify patients who may benefit from PARPi. The germline and tumor (incorporating germline and somatic) BRCA mutation testing exhibit adequate clinical validity by consistently identifying the subgroup of OC patients who benefit more from PARPi therapy and remain the gold-standard predictive biomarker for PARPi. Additionally, HRD tests using genomic scars incorporating scores of allelic imbalances (GIS or LOH) are also reasonable since this test identifies a subgroup of BRCA wild-type, platinum-sensitive cancers that will benefit from PARPi therapy in some settings [169]. However, HRD biomarkers able to evaluate cancer evolution and provide a real-time read-out of homologous recombination proficiency still need to be developed and optimized [169,183], and further studies are required to clinically validate the existing ones.

Liquid biopsy approaches may also facilitate the selection of therapy and predict chemoresistance in cancer patients by identifying mutations in genes implicated in DNA repair mechanisms. In HER+ breast cancer patients, circulating tumor DNA (ctDNA) profiling identified *ERBB2*, *TP53*, *EGFR*, *NF1*, and *SETD2* mutations contributing to trastuzumab resistance; in the same retrospective study, genetic aberrations in *TP53*, *PIK3CA*, and DNA damage repair genes were found in HER2-negative BC patients resistant to chemotherapy [184]. The components of the DNA repair machinery may also be utilized as biomarkers for evaluating tumor mutational burden (TMB). In a retrospective study of NSCLC, next-generation sequencing (NGS) was applied to different specimens, including small biopsy and cytology specimens; genomic alterations were found in genes implicated in DNA repair, including *TP53* and *BRCA2* [185]. Liquid biopsies have also been used to identify *BRCA1/2* reversions in several pathologies [186]. One example is the identification of *BRCA1/2* reversions in circulating free DNA (cfDNA) in HGOC patients treated with rucaparib [186]. In this study, 18% of platinum-resistant and 13% of platinum-refractory patients had BRCA1/2 reversions pretreatment in comparison with 2% of platinum-sensitive patients. This study supports the potential clinical use of liquid biopsies prior to initiating PARPi therapy since this test allowed the identification of patients who benefit from rucaparib therapy (patients without pretreatment cfDNA *BRCA1/2* reversions had 2-fold higher PFS on rucaparib; 9.0 versus 1.8 months; *p* < 0.001) [187]. Another advantage of liquid biopsies is the detection of clonal heterogeneity of reversion events [186]. Lin et al. described the detection of eight *BRCA1* mutation reversions in cfDNA, but only one of them was detected in the tumor biopsy [187]. The development of liquid biopsy approaches to detect specific aberrations in genes involved in DDR would provide a non-invasive and efficient means to improve treatment selection and disease outcome.

## 5. DNA Damage Response as a Mechanism of Cancer Therapy Resistance

Chemotherapy and radiotherapy rely on the cytotoxic DNA-damaging effects that for proliferating cancer cells, already burdened by genomic instability and defective DNA repair pathways, represent an induction of unrepairable genome-wide DNA damage leading to apoptosis. Therefore, chemotherapy and radiotherapy are still used as a first-line approach for many unresectable or metastatic malignancies. However, due to the large capacity of cancer cells to resist anticancer agents and adapt, the DDR is dysregulated and can lead cancer cells to genotoxic hypersensitivity or resistance development. Defective DDR allows for tumor heterogeneity development by preselecting subclones with intrinsic or acquired resistance, driving cancer progression and tumor relapse [188,189,190]. For example, in the case of cisplatin, despite its consistent rate of initial responses in multiple solid tumors [191], the treatment often results in the development of chemoresistance and therapeutic failure. Platinum salts such as cisplatin cause DNA inter- and intrastrand crosslinks, with DNA lesions repaired by a combination of NER and HR pathways. Higher expression and activity of DNA damage repair enzymes are observed in cisplatin-resistant tumor cells, and NER inhibition enhanced their sensitivity to cisplatin [192,193]. High-grade serous ovarian cancers with germline or somatic mutations in *BRCA1* or *BRCA2* genes and hypermethylation of the *BRCA1* gene and NSCLC without ERCC1 are sensitive to platinum compounds [194,195].

Temozolomide (TMZ) is the standard treatment in glioblastoma that acts mainly through O^6^-methylguanine (O^6^-meG) lesions. Other lesions caused by TMZ, such as N^3^-meA and N^7^-meG DNA adducts, are easily repaired by BER enzymes such as O-6-methylguanine-DNA methyltransferase (MGMT) [196]. The *MGMT* gene promoter hypermethylation has been associated with longer survival in glioblastoma patients treated with TMZ [197,198]. The MGMT enzyme removes alkyl groups from guanine at the O^6^ position, reducing the effect of TMZ and suggesting that MGMT activity is likely a biomarker of alkylating agents’ sensitivity [199]. On the other hand, Oldrini and colleagues (2021) reported that MGMT genomic rearrangements carried by a subset of recurrent gliomas led to MGMT overexpression and TMZ resistance in vitro and in vivo, independently from changes in its promoter methylation [200].

Radiotherapy response is also modulated by DDR machinery. In TNBC patients, low expression of 53BP1, an NHEJ pathway protein, is associated with radioresistance [201]. Glioblastoma patients with nuclear PTEN phosphorylation show reduced sensitivity to radiation by enhancing DNA repair [202]. Overexpression or activation of the BER pathway is observed in radioresistant cells. Glioma cell lines with higher endogenous APE1 endonuclease are more radioresistant, and the APE1 ectopic expression increases radioresistance [203]. Moreover, radioresistant cancer cells and biopsies from radioresistant cancer patients show low expression of GADD45α in cervical cancer [204]. Cervical cancer patients with low expression of Ku80 respond better to radiotherapy, and hypopharyngeal squamous cell carcinoma patients with low Ku70 or XRCC4 proteins have better sensitivity to chemoradiotherapy [205,206]. The residual carcinoma from patients with cervical cancer after radiotherapy showed increased expression of *DNA-PKcs*, *Ku70*, and *Ku86* genes, components of NHEJ pathways, compared to the counterpart primary tumors [207]. HR is also involved in radioresistance in cancer cells. For example, overexpression of BRCA1, BRCA2, RAD51, and RPA1 was observed in hypopharyngeal and nasopharyngeal carcinoma cells resistant to radiotherapy [208,209].

## 6. DNA Damage Response Inhibitors to Overcome Therapy Resistance

Tumor cells exhibit several therapy resistance mechanisms, but probably the most relevant ones are the inter- and intrapatient heterogeneity and intratumor heterogeneity [184]. Several strategies could be implemented to circumvent drug resistance, including adjustment of drug dose, optimization of therapies sequence, and targeting bypass mechanisms or alternative molecular targets by combinatorial approaches. The DDRi are potential alternative strategies to overcome cancer resistance (Table 2), and their combination with radiation, cytotoxic, or targeted agents can maximize the benefits of DDR targeted therapies.

### 6.1. Inhibition of PARP

In the absence of functional BRCA, targeting PARP is effective as monotherapy [210] and also sensitizes cancer cells to other drugs. In Palbociclib-resistant breast cancer cells, PARP inhibition combined with a STAT3 specific inhibitor re-sensitized cells to the agent, suggesting that concurrent targeting of DDR mechanisms and the IL6/STAT3 pathway could effectively treat acquired resistance to Palbociclib [211]. Epigenetic modifying agents have also been combined with PARP to improve therapeutic effectiveness. Specifically, the combination of DNA methyltransferase Gadecitabine and PARPi Tlazoparib were found to synergize in PARPi-resistant breast and ovarian cancer cells irrespective of BRCA status [212]. PARP inhibitors have been approved to treat high-grade serous ovarian cancer (HGSOC); however, women often develop resistance to treatment. The combination of antiangiogenic agent Cediranib with PARP inhibitor Olaparib was evaluated with varied results in a clinical trial of women with HGSOC who had developed resistance to therapy [213]. In HGSOC cell lines and PDX animal models, the checkpoint kinase 1 (CHK1) inhibitor Prexasertib showed efficacy as monotherapy but also sensitized cells to PARP inhibition [214]. PARP inhibition via Olaparib was able to reverse Sorafenib resistance in hepatocellular carcinoma by suppressing DDR mechanisms. In addition, Olaparib caused CHD1L-mediated chromatin condensation in the promoter region of transcription factors that promote cancer pluripotency [215].

Increased DNA repair in multiple myeloma is one of the main reasons for treatment resistance. Although not the standard of care for multiple myeloma, melphalan (MEL) is still used in combinatory strategies to treat this disease. To study whether PARP inhibition would reverse resistance to MEL, the agent was combined with several PARP inhibitors (Veliparib, Olaparib, and Iraparib) in multiple myeloma cell lines. The combination of MEL and PARPi in MEL-resistant cells showed an enhanced effect. However, MEL resistance, possibly caused by HR and NHEJ pathways, was not completely reversed by PARPi [216]. To discover the therapeutic potential of PARPi in combination treatment with other agents, a systems approach was developed by performing reverse-phase protein arrays to characterize adaptive responses to different therapies. The results indicate that the combination of PARPi with MEK/ERK, WEE1/ATR, and PI3K/AKT/mTOR inhibitors would show efficacy; this was also evident in various preclinical models. Based on this approach, several combinational therapies using PARPi are being assessed in clinical trials [217]. As mentioned before, TMZ, such as N^3^-meA and N^7^-meG DNA adducts, are easily repaired by BER [196]. To block BER and sensitize cells to TMZ, the agent may potentially be combined with PARP inhibitors that sustain N^3^-meA and N^7^-meG TMZ-induced lesions and improve the drug’s efficacy. A BER-independent function of PARP inhibitor Veliparib has also been shown to re-sensitize MMR deficient cells to TMZ [218]. In addition, knock-down of HR-involved proteins such as BRCA1 or RAD51 has been shown to improve the efficacy of TMZ [219].

### 6.2. Inhibition of ATM–ATR Complexes and Downstream Effectors

Resistant cancer cells may also be sensitized to treatment following exposure to ATM inhibitors. Kinases ATM, ATR, and their downstream effector kinases CHK1 and CHK2 are activated in response to DNA damage, leading to cell cycle arrest. The ATM–CHK2 axis is involved in G1 checkpoint control, whereas ATR–CHK1 controls the S and G2 checkpoints. Both ATM and ATR can convey their effects through P53, either directly or via activation of checkpoint kinase 2 (CHK2). P53 induces the CDK2 inhibitor P21 preventing damaged cells from entering the S phase [220].

Agent 2-morpholin-4-yl-6-thianthren-1-yl-pyran-4-one (KU-55933) acts as an inhibitor of ATM; specifically, it blocks phosphorylation of ATM and inhibits its downstream targets. KU55933 sensitized radioresistant breast cancer cells to ionizing radiation (IR). Specifically, breast cancer cells with a defective disabled homolog 2-interacting protein (DAB2IP) are often aggressive and resistant to radiation. KU-55933 improved the efficacy of IR against siDAB2IP breast cancer cells by targeting ATM and impairing DNA repair mechanisms [221]. Improved ATMi analogs with enhanced bioavailability, such as KU-60019 and AZ32, were also found to radio-sensitize human cancer glioma cells [222,223]. Similarly, VE-822, an ATR inhibitor, decreased the viability of pancreatic cancer cells following exposure to irradiation or to gemcitabine both in vitro and in vivo. The effect of VE-822 was achieved through dysregulation of cell cycle checkpoints and maintenance of DNA damage. Notably, the agent did not display cytotoxicity against normal cells. VE-821, another ATR inhibitor, successfully sensitized bone and ovarian cancer cells to radiation in vitro, forcing irradiated cells to divide into daughter cells and decreased survival selectively in cancer cells [224]. A detailed review describing the different approaches to sensitize cancer cells to radiation therapy by targeting DNA damage response components was recently published [225].

VX-970, a small-molecule ATR inhibitor, is currently being tested with promising results in many clinical trials in combination with chemotherapeutic drugs against resistant and aggressive cancers [226]. VX-970 also displayed radio-sensitizing effects in TNBC cells and PDX models. Specifically, the agent inhibited the ATR–CHK1–CDC25a axis signaling, sustained DNA double-strand breaks, and reduced colony formation following radiotherapy in TNBC cells. These effects were selective to cancer cells compared to normal epithelial breast cells [227]. A combination of CHK1 inhibitor PF-00477736 with Ibrutinib showed synergistic effects in vitro in several mantle cell lymphoma (MCL) cell lines. Ibrutinib is a Bruton’s tyrosine kinase (BTK) inhibitor that has been approved for refractory MCL. The study showed that in MCL cells resistant to Ibrutinib, the combination with CHK1 inhibitor led to enhanced effects [228]. The ATR inhibitor NU60 induces G2/M arrest and impairs homologous recombination, leading to increased sensitivity of breast cancer cells to DNA-damaging agents, such as cisplatin, and PARP inhibitors [229].

The APC tumor suppressor gene is inactive in 70% of sporadic breast cancers; APC-deficient tumors resemble the aggressive TNBC subtype. APC deficiency decreases sensitivity to doxorubicin (DOX), which is attributed to the inactivation of ATM, CHK1, and CHK2 and increased DNA repair in the presence of DOX. Concurrent inhibition of ATM and DNA-PK enhanced DOX-induced apoptosis in resistant cells [230]. These findings support that inhibition of the ATM–ATR–CHK axis is a promising approach to enhance radiation or chemotherapy therapeutic efficacy [231]. Importantly, synthetic lethality with ATM, ATR, and DNA-PK inhibitors is being evaluated to target HR-proficient cells [232,233].

### 6.3. Inhibition of WEE1

A recent review highlights the potential of WEE1 inhibition in radio- and chemosensitization [234]. WEE1 is a protein kinase mainly localized in the nucleus. It negatively regulates the G2/M transition following the detection of DSB [235,236]. It affects the CDK1–cyclin B complex by phosphorylating and inactivating Cyclin B on Tyr15, causing cell cycle arrest at G2. When errors happen during replication, this mechanism blocks the cell cycle to allow for repair; downregulation of WEE1, either by decreased synthesis or through proteolytic degradation, promotes entry into mitosis [237,238]. The role of WEE1 as a gatekeeper for the G2/M transition suggests that it acts as a tumor suppressor gene; however, WEE1 was overexpressed in patients with hepatocellular carcinoma, medulloblastoma, and glioma, and its levels were further elevated after exposure to chemotherapy in patients with ovarian cancer [239,240,241,242]. Overexpression of WEE1 in melanoma cells has been correlated with proliferation markers, including Ki-67 [243]. Therefore, it is postulated that the expression of WEE1 allows cancer cells to repair DNA damage following chemo- or radiotherapy, develop resistance, and continue to proliferate. In addition, cancer stem cells adopt high WEE1 expression as a protection mechanism against therapeutic agents [244]. It is well established that cancer stem cells convey resistance to DNA-damaging treatments; their percentage increases in the tumor cell population following the progressive deterioration of non-stem cells.

WEE1 inhibition represents an attractive approach for radio- and chemotherapy potentiation. Several pharmacological inhibitors belonging to different chemical classes have been developed against WEE1 and are described in a recent review [245]. AZD1775 is a WEE1 inhibitor currently in clinical trials, combined with DNA damage agents or radiotherapy. AZD1775 has been found to have a radio-sensitizing effect in pancreatic cancer, pontine gliomas, and glioblastoma [246,247,248]. Some studies suggest that AZD1775′s ability to sensitize cells to therapy is effective only in *TP53*-deficient tumors [249,250,251]. The combination of AZD1775 with cisplatin sensitized squamous cell carcinoma of the head and neck (HNSCC) cells to the latter in in vitro and in vivo models; importantly, HNSCC cells carrying high-risk *TP53* mutations became sensitive to cisplatin treatment by the selective WEE1 kinase inhibitor [252]. In conclusion, inhibition of WEE1 may sensitize cells to DNA damage therapy; although P53 has been reported to affect the effectiveness of this approach, other studies support that WEE1 inhibition sensitizes cancer cells to chemotherapeutics independently of P53 function [253].

### 6.4. Inhibition of DNA-Dependent Protein Kinase to Re-Sensitize Cells

DNA-PK belongs to the PI3K-related protein kinase (PIKK) superfamily. It participates in NHEJ to repair DSBs in DNA [254]. DNA-PK may play a role in resistance to chemotherapy and radiotherapy [255,256]. Several inhibitors, including small molecules, have been developed to target DNA-PK, block the DSB repair pathway, and sensitize cells to therapy [199,257]. The small molecule DNA-PK inhibitor, PI-103 or NU7441, combined with the third-generation epidermal growth factor receptor (EGFR) tyrosine kinase inhibitor (TKI) Osimertinib, led to synergistic effects in TKI-resistant lung cancer cells. The enhanced effect was attributed to the prolongation of DNA damage and cell cycle arrest [258]. The DNA-PKcs inhibitor NU7441 blocked glioma stem cell tumorsphere formation in vitro. In addition, in human-derived glioblastoma xenograft mice, the inhibitor blocked tumor growth and sensitized cancer cells to radiotherapy [259].

## 7. Mechanisms of Resistance to DNA Damage Response Inhibitors

**Figure 1:**
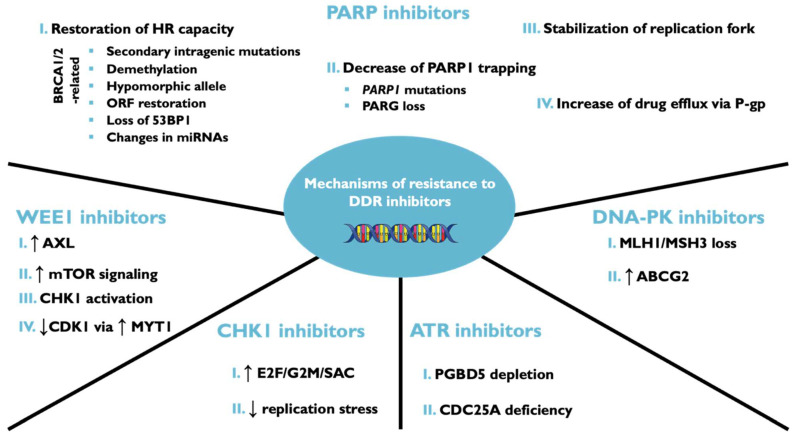
Mechanisms of resistance to DDR inhibitors. Cancer cells develop resistance to DDR through several mechanisms. The molecular mechanisms of resistance to PARPi include HR capacity restoration, decreased trapping of PARP1, stabilization of replication forks, and P-gp-mediated drug efflux. The resistance to WEE1 inhibitor is induced by AXL overexpression, mTOR signaling, CHK1 activation, and through the overexpression of MYT1 that decrease CDK1 activity. The resistance to CHK1 inhibitor is associated with increased E2F/G2M/SAC expression and reduced replication stress. The resistance to ATR inhibitor is induced by the loss of PGBD5 and CDC25A deficiency. Finally, the DNA-PK inhibitor resistance is caused by the loss of MLH1/MSH3 and the overexpression of ABCG2.

As with most target therapies, some patients are primarily resistant to DDRi and others eventually develop acquired resistance (Figure 1), with the latter being more frequent in patients with advanced disease [260]. Since PARPi are the only DDRi approved for clinical use, most known resistance mechanisms are associated with these inhibitors.

### 7.1. Resistance to PARP Inhibitors

The mechanisms of resistance to PARPi can be credited to several factors, including restoration of the mechanisms controlled by BRCA, such as HR repair and/or stabilization of replication forks [261,262]. Like other systemic chemotherapies, cancer cell develops PARPi resistance via several different mechanisms: (i) increased expression of multidrug resistance pumps (MDRs), enhancing the efflux of the PARPi out of the cell [263], (ii) reduced PARP1 binding affinity to DNA due to mutations and functional alterations of the PARP1 protein and/or disrupted PARylation [264,265], or (iii) restored HR and/or replication fork stabilization [266,267,268,269].

In BRCA-deficient tumors, the most frequent acquired resistance mechanism to PARPi is the re-establishment of BRCA1 or BRCA2 functionality by secondary intragenic mutations; specifically, genetic alterations may reinstate the open reading frame (ORF), leading to the expression of functional BRCA [270]. In addition, restoration of the wild-type BRCA protein may occur via a secondary mutation that reverses the inherited mutation or by the demethylation of the *BRCA1* promoter; both these events may lead to restoration of the wild-type BRCA protein [160,271]. Specific mutations in the BRCA gene, including the BRCA1-C61G mutation, may also confer PARPi and cisplatin resistance [272]. Another possible way of HR restoration is due to loss of the shieldin complex, consisting of REV7, c20orf196 (SHLD1), FAM35a (SHLD2), and FLJ26957 (SHLD3), which normally prevents DSB resection and facilitates NHEJ. However, if lost, shieldin can promote PARPi resistance even in the absence of BRCA [273,274]. ATPase TRIP13 inactivates the shieldin complex, triggering the 5′ to 3′ resection of double-strand breaks and promoting HR [275]. In many BRCA-deficient tumors, TRIP13 is upregulated, contributing to the intrinsic PARPi resistance. Inhibiting the ATPase domain of TRIP13 can stabilize the shieldin complex to promote NHEJ, block HR and overcome intrinsic PARPi resistance. Inhibiting TRIP13 might be useful to treat BRCA-deficient tumors with intrinsic but also acquired PARPi resistance [275]. Secondary mutations restoring BRCA function were found in patients with germline *BRCA* mutation-associated ovarian and breast cancer upon acquired resistance to PARPi and/or cisplatin [276]. Reversion mutations of *BRCA1* can also exhibit the MMEJ signature, pointing to the potential involvement of POLQ in driving resistance [277]. Consequently, inhibitors of POLQ can suppress PARPi resistance in HR and NHEJ-deficient cancers [277]. A detailed review of the mechanisms of BRCA re-activation in PARPi resistant cells was recently published [261].

In addition, *BRCA1/2*-deficient cancer cells may develop PARPi resistance by protecting their replication forks; they achieve this by blocking the recruitment of nucleases, MRE11 or MUS81, to the stalled fork, thereby resulting in fork protection [267,268]. These studies indicate that PARPi resistance is achieved without restoring HR repair. Furthermore, other mechanisms of resistance to PARPi have been reported, such as downregulation of PARP levels and increased levels of the P-glycoprotein efflux pump [260,278]. Overexpression of ABCB1 has been reported in PARPi-resistant human ovarian cancer cells; administration of MDR1 inhibitors such as Verapamil and Elacridar reversed resistance to PARPi [279]. It has been reported that most clinical PARP inhibitors induce cytotoxicity by trapping PARP1 at sites of DNA damage [280]. Resistance to PARP inhibitors can emerge through point mutations in PARP1 that alter PARP1 trapping, highlighting the importance of PARP1 intramolecular interactions in PARPi-mediated cytotoxicity [264].

### 7.2. Cell Cycle Regulators in DNA Damage Response Inhibitors in Resistant Cells

Several reports suggest that the silencing of cyclins may confer resistance to DDR inhibitors. There are two major classes of G1 cyclins that regulate cell cycle progression during the G1 phase: cyclin D, which cooperates with either CDK4 or CDK6, and cyclin E, which binds CDK2. During cell cycle progression in the early G1 phase, cyclin D–CDK4/6 complexes phosphorylate the Rb protein. Complete phosphorylation of the Rb protein is achieved at the end of the G1 phase by the CDK2/cyclin E complex. Fully phosphorylated Rb protein is inactive and releases the E2F factor, allowing the expression of S phase genes, leading the cells through the G1/S checkpoint. In response to DNA damage, p21 levels are increased; p21 binds both to the cyclin and the CDK subunits of the CDK/cyclin complex and disrupts the interaction between CDK and its substrates, blocking cell cycle progression [281].

Downregulation of cyclin D has been shown to confer resistance to CHK1 inhibition. CHK1, a serine/threonine kinase that acts as an ATM–ATR effector, is activated following exogenous DNA damage, including nicks caused by chemotherapeutic drugs. CHK1 activates the S and G2 checkpoints by controlling different mechanisms of DNA repair, including activation of homologous recombination repair or apoptosis if DNA damage is too severe [282,283]. In a MCL cell line that was made resistant to the CHK1 inhibitor PF-00477736, the re-expression of cyclin D1 partially re-sensitized cells to the agent. This suggests that low levels of cyclin D1 confer resistance to CHK1 inhibitors and that re-establishment of this protein may re-sensitize cells [284].

Cell division cycle 25A (CDC25A) is a dual-specificity phosphatase implicated in cell cycle control by inhibiting CDK phosphorylation and causing the formation of cyclin–CDK complexes. Following DNA damage, CDC25A is degraded, leading to cell cycle arrest. CDC25A is overexpressed in cancer and promotes tumorigenesis [285]; interestingly, a genome-wide CRISPR screen showed that the absence of CDC25A leads to ATR inhibitor resistance. Loss of CDC25A led to cell cycle arrest in cells treated with ATR inhibitor, diminishing the DNA damage caused by ATR inhibitors might otherwise generate; resistance was reversed using a WEE1 inhibitor that forced mitotic entry [286].

Dysfunctional apoptosis is one of the hallmarks of cancer. The increased levels and/or activity of anti-apoptotic proteins and, concurrently, the inactivation of pro-apoptotic molecules convey resistance to many anticancer drugs [287]. P53, a key molecule controlling cell cycle fate following DNA damage, is silenced in most human cancers. However, restoration of P53 following inhibition of MDM2 by Nutlin conveyed resistance to the cytotoxic effects of WEE1 inhibitor AZD1775 [288].

### 7.3. Activation of Alternative DNA Repair Pathways

Several studies report that the activation of alternative pathways to repair DNA damage is responsible for the observed resistance to DDR inhibitors. In P53-deficient cells, the induction of DSBs using a radiomimetic agent and DNA-PK inhibition led to an increased DSB burden in the S-phase; however, a subset of the cell population exhibited resistance to this combination therapy, which was caused by the recruitment of DNA polymerase theta (Pol θ or POLQ). Pol θ mediated end joining repair to improve cell viability following therapy-induced DNA damage. Concurrent inhibition of Pol θ and DNA-PK sensitized p53-deficient breast cancer cells to therapy [289].

## 8. Liabilities upon Treatment with DDR Inhibitors

Target therapies, including PARPis, contribute to important therapeutic breakthroughs in oncology, improving the quality of life and increasing the life expectancy of cancer patients. As mentioned, PARPis were demonstrated to be clinically effective, with acceptable tolerability and safety, in a specific range of solid tumors, which led to FDA and European Medicines Agency (EMA) approval of Olaparib, Rucaparib, Niraparib, and Talazoparib [31,290]. However, a consolidated body of evidence from studies of PARPi in patients has identified several adverse events and specific indications for their prevention, monitoring, and management [291,292,293,294]. PARPi display several on- and off-target toxicities, with hematological and gastrointestinal toxicities among the most common adverse events. Pneumonitis and therapy-related myeloid neoplasias (t-MN), such as AML and myelodysplastic syndromes (MDS), have been reported with PARPi, but despite their rare frequency, they are potentially life-threatening, often fatal, and deserve particular attention due to their severity [291]. The t-MN is typically a late complication of some chemo- and radiotherapy, and the subtype and latency period are usually treatment-dependent [295].

The link between PARPi and the development of t-MN is not fully understood. The pretreatment presence of clonal hematopoiesis of indeterminate potential (CHIP) with *TP53* mutations [296], a hematopoietic cell population with one or more somatic mutations/copy number alterations that can expand with time and under positive clonal selection pressures [297], have been proposed as a possible explanation. Kwan et al. also analyzed the risk of t-MN development in patients with HR gene alterations and found a higher prevalence in patients with high-grade ovarian cancer that harbored a deleterious mutation in *BRCA1*, *BRCA2*, *RAD51C*, or *RAD51D* (4.1%) compared to those with mutation-containing cancers (1.0%) and without mutations (1.0%) [296]. Mutations of DDR genes (e.g., *TP53*, *PPM1D*, and *CHEK2*) involved in CHIP occur with increased frequency in cancer patients exposed to platinum compounds/topoisomerase II inhibitors or radiation therapy [296,298]. Additionally, previous treatments with platinum and alkylating agents may increase the risk of t-MN development in BRCA-associated high-grade ovarian cancer patients treated with PARPi as maintenance therapy [299]. PARPi may potentiate t-MN in patients with preexisting CHIP by selecting clones with DDR gene mutations that improve the competitive fitness of the cells under these conditions [31,299]. Oliveira et al. (2022) performed a comprehensive analysis of the pathologic and genetic characteristics of PARPi-related t-MN patients, showing that these patients have complex karyotypes and frequently have pathogenic *TP53* mutations [300].

Most data available about t-MN arise from gynecologic cancer patients treated with Olaparib, with an estimated frequency of t-MN development between 1% in the PAOLA-1 study [105] and 8% in the SOLO-2 trial [301]. A recent study by Morice et al. (2021) evaluated the safety profile of 31 randomized controlled trials with PARPis as one arm in different tumor types and settings [302]. In this systematic review, PARP inhibitors significantly increased the risk of AML and MDS in comparison with placebo treatment (Peto OR 2.63 [95% CI 1.13–6.14], *p* = 0.026); the incidence of these t-MN across PARPi groups of 0.73% and placebo groups was 0.47%, with a median latency between first PARPi and the t-MN onset of 17.8 months [302]. The risk of t-MN development was small but more than doubled, even after controlling for prior platinum-based chemotherapy. In a meta-analysis, Nitecki et al. (2021) did not find an increased incidence of t-MN in PARPi-treated patients compared to control treatments [303]. However, patients who received a PARPi as frontline treatment and those who received fewer than two prior lines of chemotherapy showed a higher risk of t-MN [303]. In a pharmacovigilance analysis of the FDA adverse event reporting system, Ma et al. (2021) verified a dramatic increase in PARPi related t-MN from 2015 to 2019 and found a higher reporting of t-MN in patients treated with PARPi (reporting odds ratio (ROR) 16.47, 95% CI 14.72–18.44), with RORs (95% CI) of 48.03 (42.21–54.64) for Olaparib, 6.58 (5.03–8.61) for Niraparib, and 2.23 (1.32–3.77) for Rucaparib [304]. Current studies showed several limitations, including the cross-over between control and PARPi arms. This scenery may overestimate the incidence of t-MN in control/placebo arms since the subsequent therapies are not regularly reported [303].

Clinicians need to be aware of these late but potentially fatal adverse events, especially in the front-line maintenance settings, and pharmacovigilance and mechanistic studies should be implemented to improve the understanding of the risk factors that predispose to t-MN. Identifying biomarkers that discriminate patients at high risk of t-MN development upon PARPi treatment from those who benefit from frontline PARPi will improve treatment outcomes and prevent undesired adverse events.

## 9. Conclusions and Perspectives

Cancer cells display several defects in DDR pathways, offering a chance to explore these deficiencies clinically. DDR-based cancer treatments and combinatory regimens provide potential therapeutic approaches that exploit deficiency DDR pathways via synthetic lethality strategies. Despite the success of PARPi in HR-deficient cancers, such as breast, ovarian, and pancreatic cancers, several patients present serious toxicities or developed resistance to DDRi. A variety of DDRi resistance mechanisms have already been identified in preclinical models and patients, but clinical data are still scarce, and this remains an open field of research. Another challenge in DDR-based cancer treatments is the identification of genetic and functional biomarkers that define the patients who will be most suitable, suffer fewer side effects and toxicity, and benefit more from these therapeutic options. Moreover, although the higher benefits of DDRi are observed in patients with impaired DDR machinery, patients with proficient cancers can also benefit from these therapeutic approaches. Thus, further investigation is warranted to identify differential strategies for these patients, including combinatory approaches with targeted therapies such as immunotherapies (e.g., immune checkpoint inhibitors and non-specific immunotherapies), anti-angiogenic agents (e.g., VEGF inhibitors), and metabolic drugs (e.g., IDH inhibitors), among others. Another possible strategy is to combine different DDRi (e.g., PARPi with ATM, ATR, WEE1, or CHK1/2 inhibitors). Currently, PARPi is the maintenance therapy of choice for some cancers, such as ovarian, fallopian tube, primary perineal, and pancreatic cancer, showing manageable toxicity profiles. However, it should be highlighted that PARPi treatment increases the risk of AML and MDS development. This is a rare but frequently fatal event, and prescribing clinicians should remain vigilant about this complication. Additional research, including long-term pharmacovigilance studies, is needed to identify toxicity-predisposing factors and susceptibility biomarkers to further refine and personalize DDRi treatment and prevent t-MN development in front-line and maintenance settings. Furthermore, a better understanding of the molecular mechanisms of resistance to DDRi and the development of strategies to prevent or delay the acquisition of resistance are needed.

## Figures and Tables

**Table 1:** Selected DNA damage response inhibitors approved and under research.

Inhibitor	Class/Mechanisms of Action	Stage and Indication
Olaparib	PARP inhibitor	Approved: HER2-negative BC; FC; OC; PaC; PeC; PC Phase III: BC; CRC; EC; NSCLC; SCLC; SCC Phase II/III: TNBC Phase II: BlaC; CC; GC; CBM; HNC; OSC; RCC; UC; HER2-positive; Solid tumors
Talazoparib	PARP inhibitor	Approved: BC; HER2-negative BC Phase III: OC; PC Phase II: EC, FC; SCLC; SCC; TNBC; Solid tumors Phase I/II: AML
Niraparib	PARP1 inhibitor PARP2 inhibitor	Approved: FC; OC; PeC Phase III: HER2-negative BC; NSCLC; SCLC Phase II: BC; CC; CCA; CNSC; EC; GC; GBM; Glioma; HNC; Mesothelioma; NT; EsC; PaC; RCC; UC; TNBC; Solid tumors; Uveal melanoma
Rucaparib	PARP1 inhibitor PARP2 inhibitor	Approved: FC; OC; PeC; PC Phase II: BC; CC; EC; GC; UC; Mesothelioma; PaC; TNBC; Solid tumors Phase I/II: NSCLC
Pamiparib	PARP1 inhibitor PARP2 inhibitor	Approved: FC; OC; PeC (China) Phase III: Cancer Phase II: HER2-negative BC; GC Phase I/II: GBM; Glioma; Solid tumors
Veliparib	PARP inhibitor	Approved: advanced LSCC; OC; BC, LC Phase III: BC; HER2-negative BC; TNBC; NSCLC; OC Phase II/III: GBM Phase II: Brain metastases; CRC; GCEN; Malignant melanoma; PaC; RC; Solid tumors Phase I/II: Glioma; HNC; SCLC
Stenoparib	PARP1 inhibitor PARP2 inhibitorTankyrase inhibitor	Phase II: BC; OC Phase I/II: Solid tumor
Methoxyamine	APE1 inhibitor	Phase II. GBM; Mesothelioma; NSCLC Phase I/II: Solid tumors
Palbociclib	CDK4 inhibitor CDK6 inhibitor	Approved: BC Phase II/III: NSCLC Phase II: Bone metastases; Brain metastases; GIST; Mantle cell lymphoma; PaC; PC; SCC; UC Phase I/II: CRC; Malignant melanoma
Iniparib	Cell cycle inhibitor H2AFX protein stimulantTumor protein modulator	Phase II: Glioma
Ceralasertib	ATR protein inhibitor	Phase III: NSCLC Phase II: TNBC; CCA; GC; GyC; Malignant melanoma; OSC; OC; PaC; PC; SCLCN; Solid tumors Phase I/II: CLL
Elimusertib	ATR protein inhibitor	Phase I: HNC; Lymphoma; OC; Solid tumors
M 4344	ATR protein inhibitor	Phase I: Lymphoma; Solid tumors
Berzosertib	ATR protein inhibitor	Phase II: FC; LS; OC; PeC; PC; SCLC; UC; Solid tumors Phase I: HNC
AZD 1390	ATM protein inhibitor	Phase I: GBM; NSCLC; Soft tissue sarcoma Preclinical: BC; Meningioma
Prexasertib	CHEK1 inhibitor CHEK2 inhibitor	Phase II: OC; Solid tumors Phase I/II: EC; UCP reclinical: BC
SRA 737	CHEK1 inhibitor	Phase II: Solid tumors Phase I/II: OC; PC
Peposertib	DNA-PK inhibitor	Phase I/II: RC/SCLC Phase I: GBM; Cancer; Solid tumors
AZD 7648	DNA-PK inhibitor	Phase I: Soft tissue sarcoma
Adavosertib	WEE1 inhibitor	Phase II: TNBC; EC; FC; NSCLC; PeC; PC; RCC; SCLC; UtC; Solid tumors Phase I: Hematological disorders; HNC; UtC Preclinical: DLBCL; GC
Volasertib	PLK1 inhibitor	Phase III: AML Phase II: MDS; NSCLC; OC Phase I: Rhabdomyosarcoma; Solid tumors
Onvansertib	PLK1 inhibitor	Phase II: AML; PaC; PC; SCLC Phase I/II: CRC; TNBC Preclinical: CMML; Medulloblastoma; OC

AML, acute myeloid leukemia; ATM, serine protein kinase ATM; ATR, serine/threonine protein kinase ATR; BC, breast cancer; BlaC, bladder cancer; CC, cervical cancer; CCA, cholangiocarcinoma; CDK, cyclin-dependent kinase; CHEK, checkpoint kinase; CLL, chronic lymphocytic leukemia; CMML, chronic myelomonocytic leukemia; CNSC, central nerve system cancer; CRC, colorectal cancer; DLBCL, diffuse large B-cell lymphoma; DNA-PK, DNA-activated protein kinase; EC, endometrial cancer; EsC, esophageal cancer; FC, fallopian tube cancer; GBM, glioblastoma; GC, gastric cancer; GCEN, germ cell and embryonal neoplasms; GIST, gastrointestinal stromal tumor; GyC, gynecological cancer; HNC, head and neck cancer; LC, lung cancer; LS, leiomyosarcoma; LSCC, lung squamous cell carcinoma; MDS, myelodysplastic syndromes; NSCLC, non-small cell lung cancer; NT, neuroendocrine tumors; OC, ovarian cancer; OSC, osteosarcoma; PaC, pancreatic cancer; PARP, Poly(ADP-ribose) polymerase; PC, prostate cancer; PeC, peritoneal cancer; PLK1; polo-like kinase; RC; rectal cancer; RCC, renal cell carcinoma; SCC, squamous cell cancer; SCLC, small cell lung cancer; TNBC, triple-negative breast cancer; UC, urogenital cancer; UtC, uterine cancer; WEE1, WEE1-like protein kinase. ClinicalTrials.gov and AdisInsight data accessed on 20 November 2022.

**Table 2:** DNA repair pathway inhibitors currently in clinical trials to sensitize cells to therapy.

Targeting Protein	Inhibitor	Clinical Status	Disease State	NCT Number
ATR PARP	AZD6738 Olaparib	Phase 2, Recruiting	Neoadjuvant Chemotherapy-resistant residual triple-negative breast cancer	NCT03740893
PARP 1/2 and Tankyrase 1/2	2X-121	Phase 2, Active not recruiting	Metastatic breast cancer, PARPi-resistant cancer	NCT03562832
PARP	Talazoparib	Phase 2, Recruiting	Multiple cancers, patients with aberrations in DNA damage response genes	NCT04550494
PD-1 PARP	Pembrolizumab Olaparib	Phase 2, Not recruiting	Nasopharyngeal carcinoma, resistant to platinum agents	NCT04825990
Testosterone PARP	Olaparib	Phase 2, Active, not recruiting	Castration-resistant prostate cancer	NCT03516812
ATR PARP	BAY1895344 Niraparib	Phase 1, Recruiting	Advanced solid tumors, ovarian cancer	NCT04267939
PARP	Talazoparib Temozolomide	Phase 1,2 Recruiting	Metastatic castration-resistant prostate cancer and no mutations in DNA damage repair	NCT04019327
PARP	Rucaparib	Phase 2 Recruiting	Prostate cancer metastatic, resistant to androgen deprivation therapy and who carry a DNA repair gene mutation	NCT03413995
PARP	Olaparib Temozolomide IMRT	Phase 1, 2 Recruiting	Unresectable high-grade gliomas	NCT03212742

IMRT, intensity-modulated radiotherapy.
